# Genome Sequence of Lignin-Degrading Arthrobacter sp. Strain RT-1, Isolated from Termite Gut and Rumen Fluid

**DOI:** 10.1128/MRA.01442-19

**Published:** 2020-01-16

**Authors:** Matthew B. Couger, Caroline Graham, Babu Z. Fathepure

**Affiliations:** a Department of Microbiology and Molecular Genetics, Oklahoma State University, Stillwater, Oklahoma, USA; University of Southern California

## Abstract

Here, we report the genome sequence of Arthrobacter sp. strain RT-1, isolated from a cocktail of termite gut and rumen fluid. Strain RT-1 degrades a variety of lignin monomers and dimers as the growth substrates. The genome annotation predicted the genes necessary for the catabolism of lignin-derived aromatic compounds.

## ANNOUNCEMENT

Lignin is the most abundant terrestrial polymer after cellulose, ranging from 10% to 30% of the dry weight of plant biomass (1). Lignin is a heterogeneous, recalcitrant, aromatic polymer that cross-links with cellulose and hemicellulose via ester linkages and has been shown to resist microbial attack, forming a major roadblock to effective saccharification of plant polysaccharides (2, 3). In recent years, interest in finding novel bacteria that degrade lignin or lignin-derived aromatic compounds has heightened due to their metabolic versatility and their ability to grow in extreme environments; more importantly, bacteria have a simple genetic system for protein engineering that can be used to improve the performance of lignocellulose-degrading enzymes for biofuel production (4, 5).

We isolated Arthrobacter sp. strain RT-1 from a lignin-degrading enrichment developed from the gut content of termites and rumen fluid. Flasks containing mineral salts medium (MSM) and alkali lignin were inoculated with rumen fluid (2% [vol/vol]) and termite gut contents (2% [vol/vol]). Flasks were repeatedly fed with alkali lignin as the sole carbon for >12 months prior to plating the enrichment onto agar plates prepared with MSM and alkali lignin (6). A colony was picked, grown in MSM with alkali lignin, and identified as an *Arthrobacter* sp. using 16S rRNA gene sequencing (6). Genomic DNA from strain RT-1 was isolated from the culture grown on alkali lignin using the FastDNA Spin kit for soil (MP Biomedicals, Solon, OH). The library construction was done by Novogene (Beijing, China). Genomic DNA was fragmented with a restriction enzyme, and the fragments were converted into a library by end repairing, adding A to tails, purification, and PCR amplification, followed by whole-genome sequencing using an Illumina HiSeq 4000 platform by Novogene. The raw reads (3,338,128) were quality filtered with standard Illumina protocols to obtain 2,912,018  high-quality 2 × 250-bp paired-end reads. Reads containing a sequencing adapter, more than 10% ambiguous nucleotides, or a quality (Q) score less than 5 over half the read were quality filtered. The genome assembly was performed using Velvet (7) with a *k*-mer value of 55. This assembly produced 56 scaffolds with a total length of 4,545,853 bases with a GC content of 65.69%, and the final coverage was 644×. The scaffold *N*_50_ value was 282,801 bp. All gaps were determined by paired-end mapping, and the gap estimation was achieved using Velvet (7). Annotation of the genome using the Prodigal algorithm (8) produced 4,241 genes, and 4,068 of them were protein-coding genes. Pathway analysis was performed using the KEGG Automatic Annotation Server (9). The final annotation was produced by the NCBI Prokaryotic Genome Annotation Pipeline.

The genome analysis revealed the presence of putative genes responsible for the degradation of lignin and lignin-derived aromatic compounds, including many ring-oxidizing genes, ring-cleaving genes, and genes that code for the catechol and protocatechuate branches of the β-ketoadipate pathway (10–13). These findings indicate the potential role of strain RT-1 in lignocellulosic biomass conversion to biofuel.

### Data availability.

This whole-genome sequence has been deposited in DDBJ/EMBL/GenBank under the accession number QRGQ00000000, BioProject accession number PRJNA484028, and SRA accession number SRR10527363.
